# A zebrafish model of inflammatory lymphangiogenesis

**DOI:** 10.1242/bio.013540

**Published:** 2015-09-14

**Authors:** Kazuhide S. Okuda, June Pauline Misa, Stefan H. Oehlers, Christopher J. Hall, Felix Ellett, Sultan Alasmari, Graham J. Lieschke, Kathryn E. Crosier, Philip S. Crosier, Jonathan W. Astin

**Affiliations:** 1 Department of Molecular Medicine & Pathology, School of Medical Sciences, University of Auckland, Auckland 1142, New Zealand; 2 Department of Molecular Genetics and Microbiology, Duke University Medical Center, Durham 27710, USA; 3 Australian Regenerative Medicine Institute, Monash University, Clayton, Victoria 3800, Australia

**Keywords:** Zebrafish, Inflammation, Lymphatic, Inflammatory bowel disease

## Abstract

Inflammatory bowel disease (IBD) is a disabling chronic inflammatory disease of the gastrointestinal tract. IBD patients have increased intestinal lymphatic vessel density and recent studies have shown that this may contribute to the resolution of IBD. However, the molecular mechanisms involved in IBD-associated lymphangiogenesis are still unclear. In this study, we established a novel inflammatory lymphangiogenesis model in zebrafish larvae involving colitogenic challenge stimulated by exposure to 2,4,6-trinitrobenzenesulfonic acid (TNBS) or dextran sodium sulphate (DSS). Treatment with either TNBS or DSS resulted in vascular endothelial growth factor receptor (Vegfr)-dependent lymphangiogenesis in the zebrafish intestine. Reduction of intestinal inflammation by the administration of the IBD therapeutic, 5-aminosalicylic acid, reduced intestinal lymphatic expansion. Zebrafish macrophages express vascular growth factors *vegfaa*, *vegfc* and *vegfd* and chemical ablation of these cells inhibits intestinal lymphatic expansion, suggesting that the recruitment of macrophages to the intestine upon colitogenic challenge is required for intestinal inflammatory lymphangiogenesis. Importantly, this study highlights the potential of zebrafish as an inflammatory lymphangiogenesis model that can be used to investigate the role and mechanism of lymphangiogenesis in inflammatory diseases such as IBD.

## INTRODUCTION

Crohn's disease and ulcerative colitis are characterised as chronic inflammatory disorders of the gastrointestinal tract and together are known as inflammatory bowel disease (IBD). It is well accepted that the dysregulation of normally controlled immune responses to gut microbiota, as well as genetic susceptibility and other environmental factors contribute to the pathogenesis of IBD (Zhang and Li, 2014), however the aetiology of IBD is still not well understood.

Increased intestinal lymphatic vessel density is observed in IBD patients (Fogt et al., 2004; Geleff et al., 2003; Kaiserling et al., 2003; Pedica et al., 2008; Rahier et al., 2011) and in mouse models of IBD (D'Alessio et al., 2014; Ganta et al., 2010; Jurisic et al., 2013). Although increased lymphangiogenesis is proposed to occur in response to the decreased mesenteric lymphatic function observed in IBD patients (Heatley et al., 1980; Van Kruiningen and Colombel, 2008; Van Kruiningen et al., 2014), the mechanism by which these lymphatic vessels form is still unclear. The growth of lymphatic vessels during early lymphatic development largely involves the sprouting of lymphatic endothelial cells from the veins (Srinivasan et al., 2007; Yaniv et al., 2006) and requires vascular endothelial growth factor 3 (VEGFR-3) signalling (Irrthum et al., 2000; Karkkainen et al., 2000, 2001; Veikkola et al., 2001). The binding of extracellular VEGF ligands, in particular VEGF-C and VEGF-D, activates VEGFR-3 (Karkkainen et al., 2004; Kukk et al., 1996). Reducing inflammatory lymphangiogenesis using a VEGFR-3 blocking antibody, or decreasing lymphatic function through the down regulation of *FoxC2*, a gene required for lymphatic homeostasis, significantly increases inflammatory oedema and inhibits disease resolution in mouse models of IBD, suggesting a protective role of intestinal lymphatics in IBD (Becker et al., 2015; Jurisic et al., 2013). In agreement, a recent study has shown that enhancing lymphangiogenesis and lymphatic function, by adenoviral induction of VEGF-C, alleviates experimental chronic intestinal inflammation by increasing inflammatory cell mobilisation and bacterial antigen clearance (D'Alessio et al., 2014). Although these studies suggest there is potential in treating IBD by improving intestinal lymphatic function, further studies are required to determine the mechanism of IBD-associated lymphangiogenesis.

While the VEGF-C/VEGFR-3 signalling pathway has been associated with mammalian IBD-associated lymphangiogenesis (D'Alessio et al., 2014; Jurisic et al., 2013), the significance of other VEGFR signalling pathways implicated in inflammatory lymphangiogenesis, such as VEGF-A/VEGFR-2 (Cursiefen et al., 2004; Halin et al., 2007; Kataru et al., 2009; Kunstfeld et al., 2004; Tan et al., 2013) and VEGF-D/VEGFR-3 (Baluk et al., 2005; Huggenberger et al., 2010; Kataru et al., 2009; Tan et al., 2013) is still undetermined. In addition, the cells and tissues providing the pro-lymphatic VEGFs that mediate IBD-associated lymphangiogenesis remain to be identified. In IBD patients, increased levels of lymphatic growth factors VEGF-A, VEGF-C, and VEGF-D have been reported in serum and mucosa culture supernatants (Algaba et al., 2013; Bousvaros et al., 1999; D'Alessio et al., 2014; Duenas Pousa et al., 2007; Kanazawa et al., 2001; Pousa et al., 2008). Peripheral blood mononuclear cells (Griga et al., 1999a,c), intestinal mucosa (Griga et al., 1999a, 2002), adipose tissue (Schaffler et al., 2006), and fibroblasts (Beddy et al., 2004) have all been suggested to provide VEGF-A in IBD and may be particularly important for IBD-associated angiogenesis. However, the cellular sources of VEGF-C (and VEGF-D) required for IBD-associated lymphangiogenesis are still not clear.

In other inflammatory situations such as chronic airway inflammation, skin inflammation, keratitis, and peritonitis, macrophages express pro-lymphatic growth factors, such as VEGF-A, VEGF-C, and VEGF-D, and macrophage depletion results in reduced inflammatory lymphangiogenesis (Baluk et al., 2005; Cursiefen et al., 2004; Harvey and Gordon, 2012; Kataru et al., 2009; Kim et al., 2009; Kubota et al., 2009; Maruyama et al., 2005; Tan et al., 2014; Zhang et al., 2007). A recent study has shown that neutrophils can stimulate skin inflammation-associated inflammatory lymphangiogenesis by increasing VEGF-A bioavailability, and also by secreting VEGF-D (Tan et al., 2013). Intestinal epithelial cells have also been shown to express VEGF-C (Joory et al., 2006). Therefore, it is possible that macrophages, neutrophils, and intestinal epithelial cells may contribute to IBD-associated lymphangiogenesis by secreting VEGF-A, VEGF-C, and VEGF-D. The availability of zebrafish transgenic lines that mark lymphatic vessels (Gordon et al., 2013; Okuda et al., 2012; van Impel et al., 2014), leukocytes (Ellett et al., 2011; Hall et al., 2007), and the intestinal epithelial cells (Her et al., 2004) makes the zebrafish model an ideal platform to uncover the *in vivo* interaction between these cell populations and lymphatic vessels in IBD.

Zebrafish larvae treated with the colitogenic agents 2,4,6-trinitrobenzenesulfonic acid (TNBS) or dextran sodium sulphate (DSS) develop intestinal inflammation with IBD-like characteristics including: (1) the requirement for microbiota to trigger inflammation; (2) responsiveness to anti-inflammatory medications; (3) increased expression of pro-inflammatory cytokines; (4) increased leukocyte recruitment to the intestine; (5) enlarged intestinal lumen and smoothening of microvilli (Fleming et al., 2010; Oehlers et al., 2012, 2011; Yang et al., 2014). However, as the zebrafish are immersed in the colitogenic agents, inflammation is not restricted to the intestine in this IBD-model.

Zebrafish are also gaining standing as a model for lymphatic development and, importantly, the requirement for Vegfr3/Vegfc signalling in lymphatic development is conserved in zebrafish (Hogan et al., 2009; Kuchler et al., 2006; Le Guen et al., 2014; Okuda et al., 2012; Yaniv et al., 2006). Combining existing zebrafish IBD and lymphatic models may therefore provide a novel platform for IBD-associated lymphangiogenesis research. In this study, we show that intestinal inflammation triggered by exposure of zebrafish larvae to TNBS or DSS resulted in Vegfr-dependent lymphangiogenesis, specifically in the zebrafish intestine. Furthermore, we show that zebrafish leukocytes express vascular growth factors and that macrophages are required for inflammatory lymphangiogenesis following colitogenic challenge.

## RESULTS

### Colitogenic challenge using TNBS or DSS stimulates development of lymphatic sprouts in the intestine

The aim of this study was to establish an inflammatory lymphangiogenesis model in zebrafish. To do this, we utilised the *lyve1:DsRed2;kdrl:EGFP* compound transgenic line which labels both the lymphatic/venous (*lyve1*) and blood (*kdrl*) vasculature and can therefore be used to differentiate the lymphatic vessels from the blood vessels (Okuda et al., 2012). Previous characterisation of this line has revealed that zebrafish develop an intestinal lymphatic (IL) network (summarised in supplementary material Fig. S1) (Okuda et al., 2012). Intestinal lymphatic sprouts (ILSs) (previously described as lymphatic branches) grow between the major intestinal lymphatic vessels and are rare on the left side of the zebrafish intestine at 7 days post-fertilisation (dpf). From 7 dpf, the number and length of the ILS steadily increases until 15 dpf, where they are hypothesised to contribute to the web-like IL that forms across the intestinal bulb (Okuda et al., 2012). The lack of ILSs on the left side of the larvae at 7 dpf provides a physiologically-relevant opportunity to identify and quantify intestinal lymphatic network expansion in intestinal disease.

**Fig. 1.**
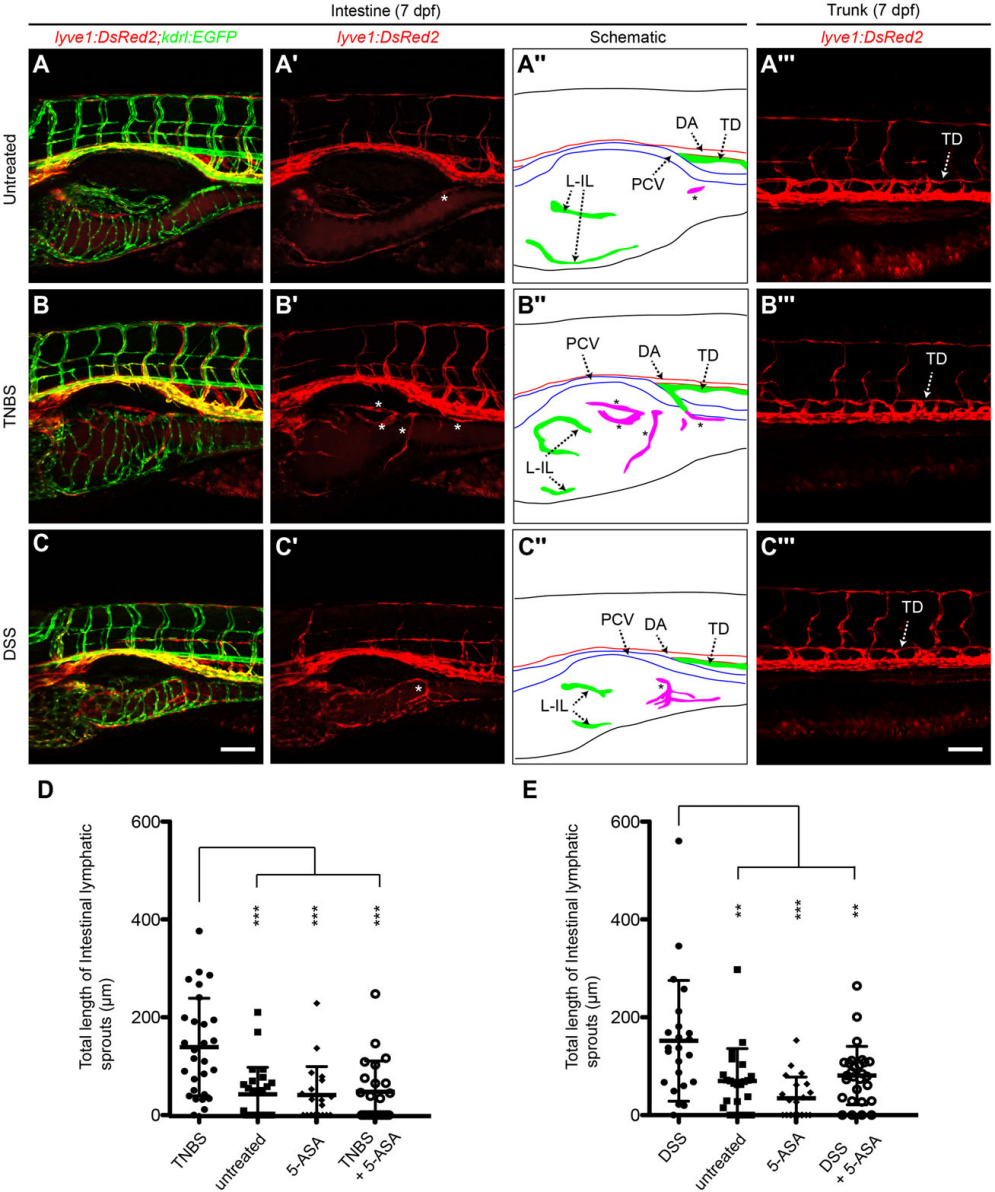
**Colitogenic challenge is associated with increased intestinal lymphangiogenesis.** (A-C) Lateral images of *lyve1:DsRed2;kdrl:EGFP* larvae at 7 dpf (A), treated with TNBS (B) or DSS (C). Asterisks indicate intestinal lymphatic sprouts (ILSs). A′-C′ shows the left intestinal vasculature and A‴-C‴ shows the trunk vasculature in the DsRed channel only. A″-C″ are schematic diagrams of arteries (red), veins (blue), lymphatic vessels (green) and ILS (magenta) of A-C. (D-G) Quantification of ILS number (D,E) and total ILS length (F,G) in TNBS (D,F) and DSS (E,G) treated larvae compared with untreated, 5-aminosalicylic acid (5-ASA), and TNBS/DSS+5-ASA (*n*≥20). Error bars, ±s.d. *P<0.05, **P<0.01, ***P<0.001 by one-way ANOVA with Dunnett's multiple comparison test. DA, dorsal aorta; L-IL, left intestinal lymphatics; PCV, posterior cardinal vein; TD, thoracic duct. Scale bar: 100 μm.

When intestinal inflammation was induced using either TNBS or DSS in 3 dpf *lyve1:DsRed2;kdrl:EGFP* embryos, the number and total length of the ILSs were increased at 7 dpf when compared with untreated larvae (Fig. 1). Increased lymphangiogenic activity was intestine-specific, as no ectopic lymphatic vessels were observed in the trunk (Fig. 1A‴-C‴) or the head (data not shown). The lymphatic vessels induced following TNBS or DSS treatment predominately grew over the outer surface of the intestinal epithelium. However, there were rare examples where these ILS grew towards the swim bladder, dorsal to the intestine. Blood vessel development in the intestine also appeared normal in TNBS and DSS-treated larvae (Fig. 1A-C). To show that the increased ILS formation was inflammation-dependent, we suppressed inflammation in TNBS or DSS treated larvae by co-administration of the anti-inflammatory drug 5-aminosalicylic acid (5-ASA) which is used to treat IBD (Rutgeerts et al., 2009). With 5-ASA co-treatment, the number and total length of the ILSs was reduced when compared with larvae exposed to colitogenic agents alone (Fig. 1D-G), showing that TNBS/DSS-driven lymphangiogenesis in the intestine is associated with inflammation. We therefore termed this model zebrafish intestinal inflammatory lymphangiogenesis (IIL).

### 5-ASA reduces neutrophil and macrophage recruitment to the intestine

**Fig. 2.**
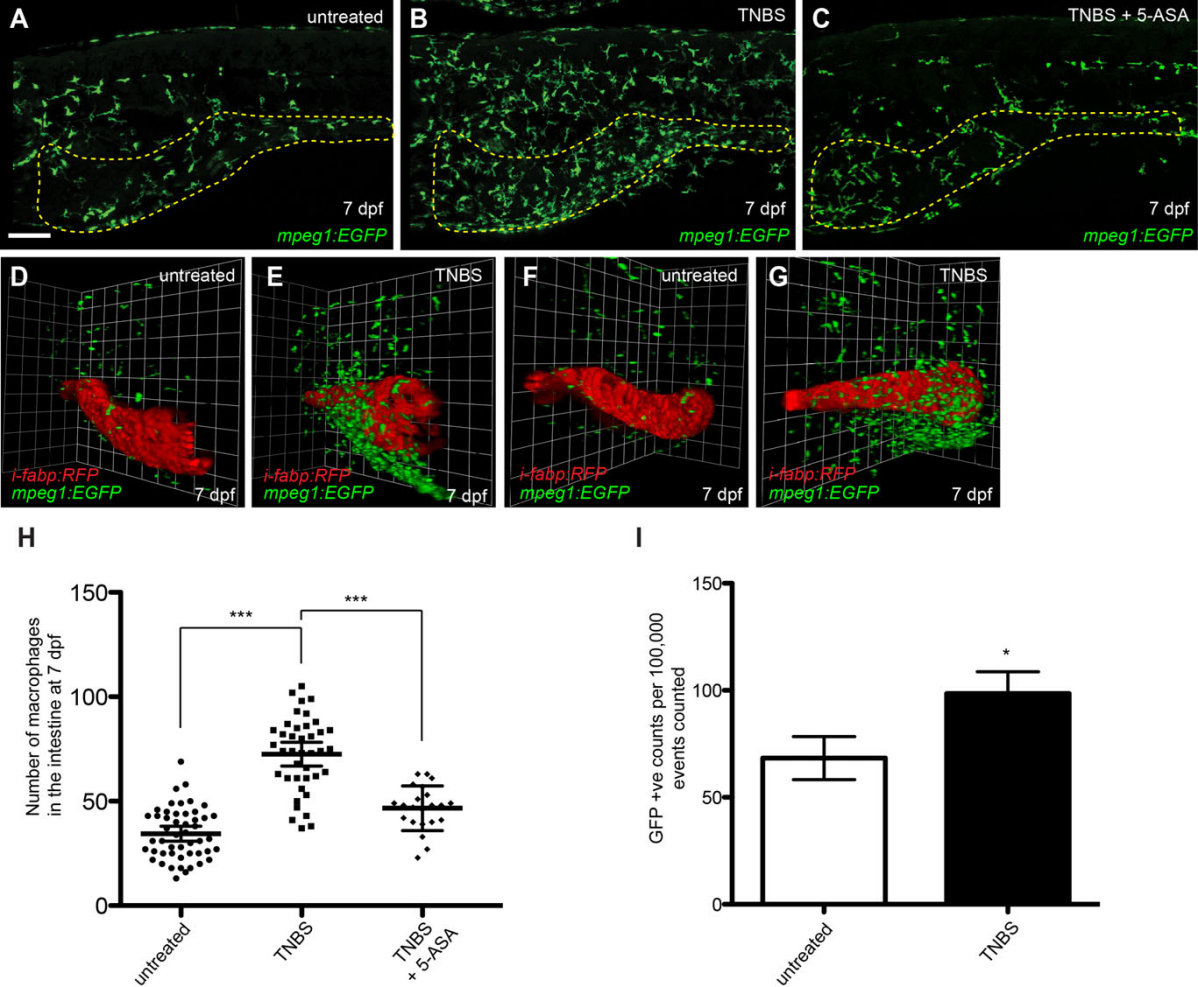
**Treatment with 5-aminosalicylic acid (5-ASA) reduces TNBS-mediated macrophage recruitment to the intestine.** (A-C) Lateral images of the intestine of untreated (A), TNBS-treated (B) and TNBS+5-ASA (C) *mpeg1:EGFP* larvae at 7 dpf. The intestine is outlined with a yellow dotted line. (D-G) Three-dimensional reconstruction of the intestine in untreated (D,F) and TNBS-treated (E,G) *mpeg1:EGFP;i-fabp:RFP* larvae at 7 dpf showing increased recruitment of *mpeg1-*expressing macrophage lineage cells to *i-fabp*-expressing intestinal epithelial cells following TNBS treatment. D,E and F,G represent two different angles taken from the same animal. (H) Quantification of macrophages in the intestine of TNBS-treated larvae compared with untreated and TNBS+5-ASA at 7 dpf (*n*≥22). (I) FACS analysis of EGFP-positive cell counts from untreated and TNBS-treated 7 dpf *mpeg1:EGFP* larvae (*n*≥3, each replicate contains 30–40 larvae). Error bars, ±s.d. *P<0.05, ***P<0.001, by one-way ANOVA with Dunnett's multiple comparison test (H) or unpaired *t*-test (I). Scale bars: 100 μm.

Given that macrophages and neutrophils are known to contribute to inflammatory lymphangiogenesis in mammalian models (Tan et al., 2014), we next investigated whether reduction of zebrafish IIL following 5-ASA treatment was associated with decreased immune cell recruitment to the intestine. Using the neutrophil-specific *lysozyme C* promoter driven *Tg(lyz:EGFP)* transgenic line (Brannon et al., 2009; Hall et al., 2007) we confirmed our previous study showing that TNBS exposure results in an increase in neutrophil recruitment to the zebrafish intestine and that treatment with 5-ASA suppresses this (Oehlers et al., 2011) (see supplementary material Fig. S2). To establish whether TNBS treatment also altered macrophage recruitment to the intestine, larvae expressing the macrophage-specific *macrophage expressed gene 1* promoter driven *Tg(mpeg1:EGFP)* transgene (Ellett et al., 2011) were treated at 3 dpf with TNBS and the number of *mpeg1*-positive, macrophage-lineage cells in the intestine assessed at 7 dpf. The number of macrophages in the intestine of TNBS-treated larvae increased 2.2-fold when compared with untreated larvae (untreated, 34±12; TNBS-treated, 73±18) (Fig. 2A,B,H). Confocal live imaging of double transgenic *mpeg1:EGFP;intestinal fatty acid binding protein (i-fabp):RFP* (Her et al., 2004) larvae demonstrated that macrophages accumulated around the outer surface of the *i-fabp*-expressing intestinal epithelial cells following TNBS treatment (Fig. 2D-G). The total number of EGFP-positive macrophages in *mpeg1:EGFP* larvae, quantified by fluorescence activated cell sorting (FACS), increased 1.5-fold following TNBS treatment (untreated, 68/100,000±10; TNBS-treated, 99/100,000±10) (Fig. 2I). From this data we conclude that TNBS induces a systemic inflammatory response that is particularly severe in the intestine. Finally, 5-ASA suppressed the recruitment of macrophages to the zebrafish intestine in larvae exposed to TNBS; larvae treated with 5-ASA+TNBS had less intestinal macrophages when compared with TNBS-treated larvae (Fig. 2A-C,H). Taken together these results show that 5-ASA co-treatment reduces TNBS-mediated inflammation in the zebrafish intestine.

### Zebrafish macrophages and neutrophils express lymphatic growth factors

**Fig. 3.**
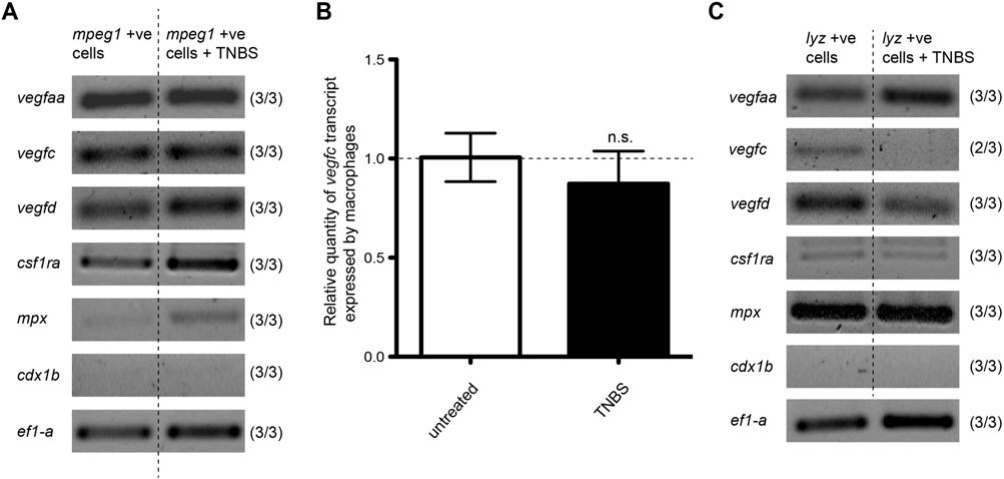
**Zebrafish macrophages and neutrophils express lymphatic growth factors.** (A) RT-PCR of *vegfaa*, *vegfc*, *vegfd*, *csf1ra*, *mpx*, *cdx1b*, and *ef1-a* from RNA isolated from untreated and TNBS-treated 7 dpf zebrafish macrophage-lineage cells. (B) qPCR analysis of *vegfc* mRNA in TNBS-treated 7 dpf zebrafish macrophages relative to untreated 7 dpf zebrafish macrophages (*n*=3). (C) RT-PCR of *vegfaa*, *vegfc*, *vegfd*, *csf1ra*, *mpx*, *cdx1b*, and *ef1-a* from RNA isolated from untreated and TNBS-treated 7 dpf zebrafish neutrophils. The colours in the gel images are inverted and the numbers in brackets indicate reproducibility over three experiments. Error bars, ±s.d. n.s. *P*>0.05 by unpaired *t*-test.

RT-PCR was used to investigate whether zebrafish larval macrophages express lymphatic growth factors. Macrophage-lineage cells were isolated from zebrafish larvae by FACS of EGFP-positive cells from the *mpeg1:EGFP* transgenic at 7 dpf. The isolated population was subsequently confirmed to be macrophages as cells strongly expressed the macrophage marker, colony stimulating factor 1 receptor a (*csf1ra*) with little or no expression of the neutrophil marker, myeloperoxidase (*mpx*) or the intestinal marker, caudal type homeobox 1 b (*cdx1b*) (Fig. 3A) (Flores et al., 2008; Meijer et al., 2008). We found that zebrafish macrophage-lineage cells expressed *vegfaa*, *vegfc*, and *vegfd* in both untreated and TNBS-treated larvae (Fig. 3A). A previous study has shown that the expression level of *vegfc* in macrophages increases in a mouse model of skin inflammation, contributing to the increased VEGF-C concentration in the inflamed site (Kataru et al., 2009). To investigate if this occurs in zebrafish IBD, the expression level of *vegfc* in zebrafish macrophages isolated from TNBS-treated larvae was compared with that of untreated larvae using qPCR and was shown to remain unchanged (Fig. 3B). To investigate whether zebrafish neutrophils express lymphatic growth factors, zebrafish neutrophils were isolated by collecting EGFP-positive cells from the *lyz:EGFP* transgenic using FACS at 7 dpf. The isolated cell population strongly expressed *mpx*, with little or no expression of *csf1ra* and *cdx1b* (Fig. 3C), consistent with a neutrophil phenotype. Zebrafish neutrophils were found to express *vegfaa* and *vegfd* in both untreated and TNBS-treated larvae, however, we did not consistently obtain an RT-PCR product for *vegfc* (Fig. 3C), suggesting that zebrafish neutrophils may only express low levels of *vegfc* mRNA.

### Zebrafish intestinal epithelial cells express lymphatic growth factors

Epithelial cells are a source of VEGF-A (Gong and Koh, 2010; Halin et al., 2007) and VEGF-C (Baluk et al., 2005) in mouse models of inflammatory diseases. We therefore hypothesised that intestinal epithelial cells may also be a source of lymphatic growth factors in our zebrafish IIL model. To investigate this, RFP-positive cells were isolated by FACS from the *i-fabp:RFP* transgenic line (Her et al., 2004), at 7 dpf, and the expression of lymphatic growth factors was studied using RT-PCR. The isolated cell population strongly expressed the intestinal marker *cdx1b*, with little or no expression of the leukocyte markers *csf1ra* and *mpo,* (see supplementary material Fig. S3), consistent with an intestinal epithelial phenotype. Zebrafish intestinal epithelial cells from both untreated and TNBS-treated larvae expressed both *vegfaa* and *vegfc* (see supplementary material Fig. S3). However, there was no difference in the expression levels of *vegfc* and *vegfaa* in zebrafish intestinal epithelial cells isolated from untreated and TNBS-treated larvae (see supplementary material Fig. S3), suggesting they are unlikely to have a major role in IIL.

### The Vegfr pathway is required for zebrafish intestinal inflammatory lymphangiogenesis

**Fig. 4.**
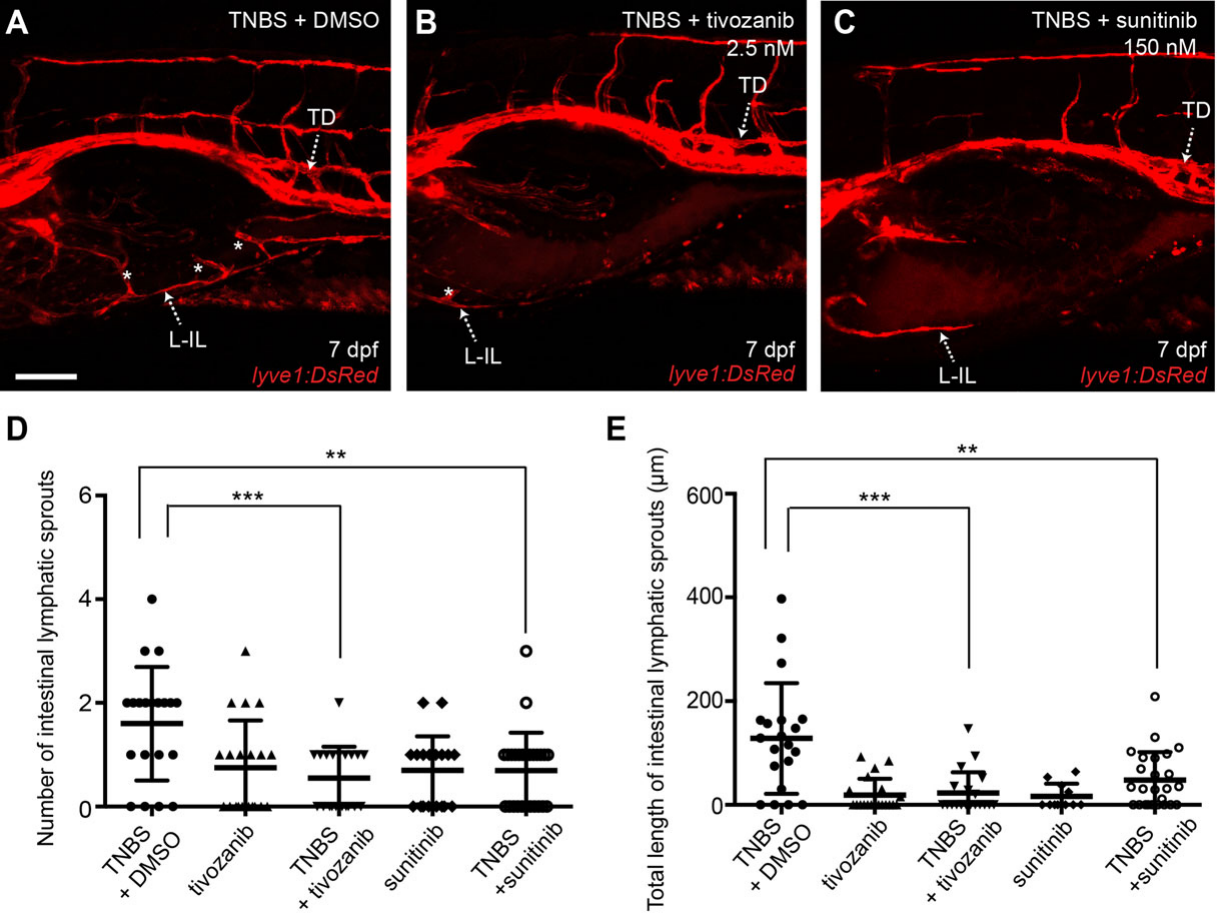
**The Vegfr pathway is required for zebrafish intestinal inflammatory lymphangiogenesis.** (A-C) Lateral images of intestine in 7 dpf *lyve1:DsRed2* larvae exposed to TNBS+1% DMSO (A), TNBS+2.5 nM tivozanib (B) and TNBS+150 nM sunitinib (C). Asterisks indicate intestinal lymphatic sprouts (ILSs). (D,E) Quantification of ILS number (D) and total ILS length (E) in TNBS+1% DMSO treated larvae compared with 2.5 nM tivozanib, TNBS+2.5 nM tivozanib, 150 nM sunitinib and TNBS+150 nM sunitinib treated larvae (*n*≥20). Error bars, ±s.d. **P<0.01, ***P<0.001 by one-way ANOVA with Dunnett's multiple comparison test. L-IL, left intestinal lymphatics; TD, thoracic duct. Scale bar: 100 μm.

As zebrafish macrophages and neutrophils express *vegfaa/c/d*, we next investigated whether the Vegfr pathway is required for zebrafish IIL. Two pan-VEGFR inhibitors were used: tivozanib (Buchanan et al., 2012; Nakamura et al., 2006) or sunitinib (Cao et al., 2008; Mendel et al., 2003). The efficacy of these inhibitors on lymphatic vessel development was determined by treating 3 dpf embryos with either 50 nM tivozanib or 3 μM sunitinib, which resulted in a lack of trunk and intestinal lymphatics at 7 dpf (see supplementary material Fig. S4). In our model, the ILS generated during IIL develop from the intestinal lymphatics, therefore we needed to reduce the dose of each inhibitor to allow the development of the intestinal lymphatic vessels. We chose to use a 20-fold lower dose of each inhibitor (2.5 nM tivozanib or 150 nM of sunitinib) as these doses were not sufficient to inhibit developmental lymphangiogenesis (Fig. 4A-E, see supplementary material Fig. S4). Co-administration of either Vegfr inhibitor with TNBS to 3 dpf embryos resulted in significantly reduced IIL at 7 dpf when compared with the 1% DMSO and TNBS co-treatment vehicle control (Fig. 4A-E) suggesting that the Vegfr pathway is required for IIL in zebrafish.

### Macrophages are required for zebrafish intestinal inflammatory lymphangiogenesis

**Fig. 5.**
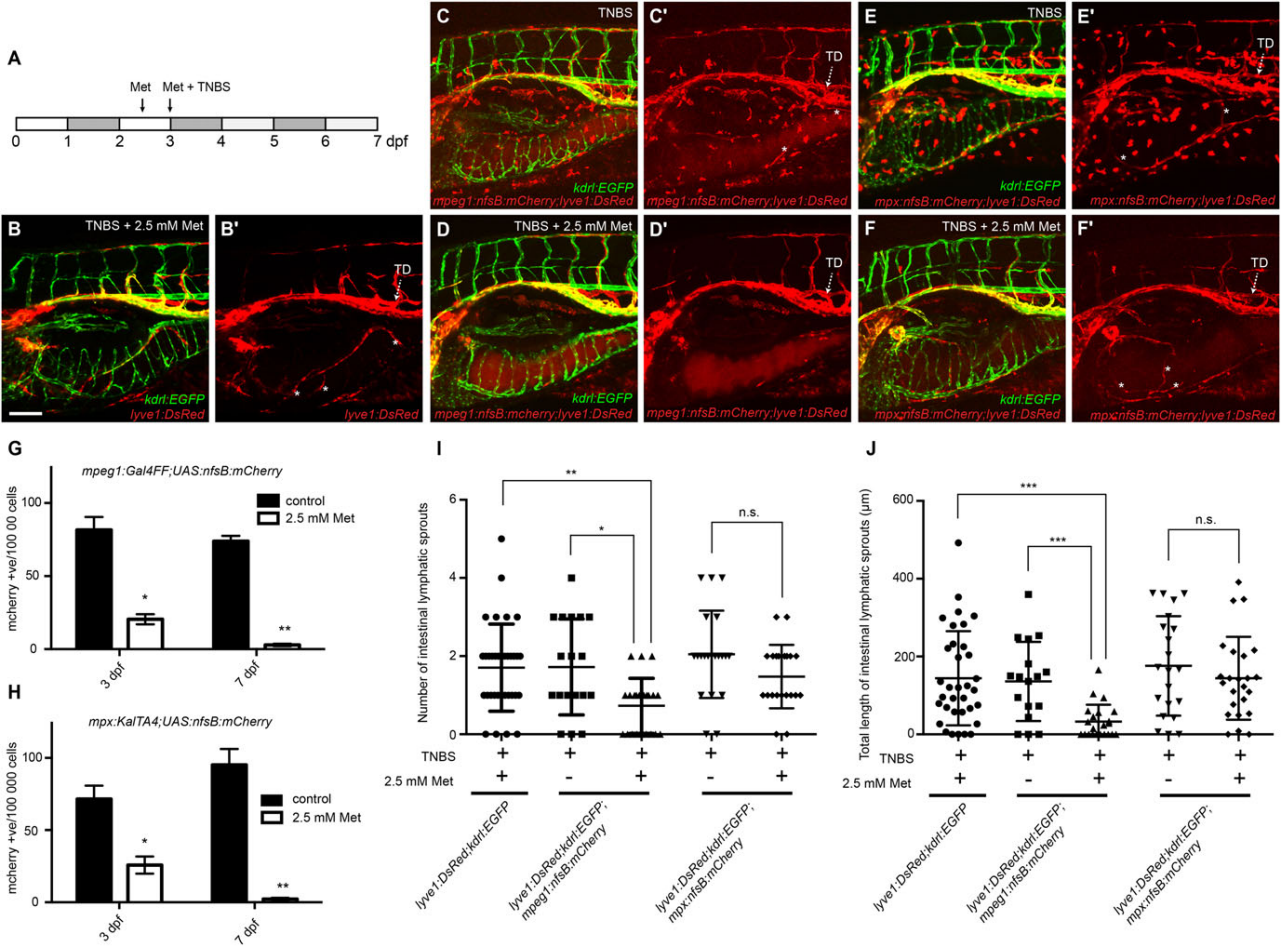
**Macrophages are required for intestinal inflammatory lymphangiogenesis.** (A) Schema outlining metronidazole and TNBS treatments. (B) Lateral image of the intestine in 7 dpf *lyve1:DsRed2; kdrl:EGFP* larvae exposed to TNBS+2.5 mM metronidazole (Met). (C,D), Lateral image of the intestine in 7 dpf *lyve1:DsRed2;kdrl:EGFP;mpeg1:Gal4FF;UAS:nfsB:mCherry* larvae exposed to TNBS (C) and TNBS+2.5 mM metronidazole (D). (E,F), Lateral image of the intestine in 7 dpf *lyve1:DsRed2;kdrl:EGFP;mpx:KalTA4;UAS:nfsB:mCherry* larvae exposed to TNBS (E) and TNBS+2.5 mM metronidazole (F). (B′-F′) shows expression in the DsRed/mCherry channel only. Asterisks indicate intestinal lymphatic sprouts (ILSs). (G,H) FACS analysis of mCherry-positive cell counts from TNBS-treated (control) and TNBS+2.5 mM metronidazole-treated 3 and 7 dpf *mpeg1:Gal4FF;UAS:nfsB:mCherry* larvae (G) or *mpx:KalTA4;UAS:nfsB:mCherry* larvae (H) (*n*≥3, each replicate contains 30–80 larvae). (I,J) Quantification of ILS number (I) and total ILS length (J) (*n*≥17). Error bars, ±s.d. n.s. *P*>0.05, *P<0.05, **P<0.01, ***P<0.001, by unpaired *t*-test (G,H) or by one-way ANOVA with Tukey's multiple comparison test (I,J). TD, thoracic duct. Scale bar: 100 μm.

To determine whether macrophages and/or neutrophils are required for IIL we utilised transgenic lines in which either macrophage-lineage cells (*mpeg1:Gal4FF;UAS:nfsB:mCherry*) or neutrophils (*mpx:KalTA4;UAS:nfsB:mCherry*) co-express mCherry and the bacterial enzyme nitroreductase *(nfsB)*. Following addition of metronidazole, nitroreductase-expressing cells are able to convert this pro-drug into a cytotoxic metabolite resulting in cell death (Curado et al., 2007; Pisharath et al., 2007). Compound *lyve1:DsRed;kdrl;EGFP;mpeg1:Gal4FF;UAS:nfsB:mCherry* and *lyve1:DsRed;kdrl;EGFP;mpx:KalTA4;UAS:nfsB:mCherry* transgenic lines were pre-treated with 2.5 mM metronidazole at 2.3 dpf, before exposure to a TNBS and metronidazole co-treatment from 3–7 dpf (Fig. 5A). This treatment regimen, achieved a 75% reduction in macrophage-lineage cells and a 65% reduction in neutrophils by 3 dpf and at 7 dpf, the numbers of both cell types were reduced by over 95% (Fig. 5G,H). IIL was significantly reduced following the ablation of *mpeg1*-expressing cells (Fig. 5C,D,I,J) but was not affected by the ablation of *mpx*-expressing cells (Fig. 5E,F,I,J) suggesting that macrophages but not neutrophils mediate inflammatory lymphangiogenesis in the zebrafish intestine following treatment with TNBS. Importantly, when *lyve1:DsRed;kdrl;EGFP* animals that did not express nitroreductase were exposed to 2.5 mM metronidazole and TNBS, we observed normal levels of IIL, showing that metronidazole alone does not inhibit inflammatory lymphangiogenesis (Fig. 5B,I,J). As haemogenic endothelium-derived cells have been implicated in developmental lymphangiogenesis (Klotz et al., 2015; Stanczuk et al., 2015) one explanation for the lack of IIL following macrophage ablation is that they are required for non-inflammatory, developmental lymphangiogenesis that is required to generate the parent intestinal lymphatic vessels for IIL to occur. To test this, we measured thoracic duct formation at 5 dpf and ILS length at 7 dpf following the ablation of macrophages and found that both were normal when *mpeg1*-expressing cells were ablated in embryos that were not treated with TNBS (see supplementary material Fig. S5), suggesting that these cells are not required for normal lymphatic development in zebrafish and that macrophages have a specific role in inflammatory lymphangiogenesis.

## DISCUSSION

In this study, we have shown that TNBS and DSS-induced colitogenic challenge results in increased ILS development, which we termed zebrafish IIL. We confirmed that zebrafish IIL was inflammatory-driven, as reduction of intestinal inflammation using the IBD therapeutic anti-inflammatory 5-ASA suppressed IIL. These results demonstrate that zebrafish exposed to DSS or TNBS can model the increased intestinal lymphangiogenesis observed in IBD patients (D'Alessio et al., 2014; Fogt et al., 2004; Geleff et al., 2003; Kaiserling et al., 2003; Pedica et al., 2008; Rahier et al., 2011). We could not determine if the increase in ILS number and length following TNBS/DSS treatment was due to ectopic ILS formation or from accelerated development of normal ILS formation; nevertheless, this is the first report demonstrating that zebrafish lymphatic vessels can respond to inflammatory stimulation and establishes a zebrafish model to study inflammatory-driven lymphatic growth. A recent study in mice has shown that lymph node lymphangiogenesis in response to oxazolone-induced inflammation can be visualised using a Prox1 lymphatic reporter line (Truman et al., 2012, 2013). However, our zebrafish model of inflammatory lymphangiogenesis enables us to visualise the interactions between leukocytes, inflamed tissues, and lymphatic vessels in response to inflammatory stimuli in a live embryo. Furthermore, the optical transparency of zebrafish embryos allows time-lapse imaging of developing inflammatory-associated lymphatic vessels at single-cell resolution, which is currently still challenging in adult zebrafish and in mammalian models of IBD.

While our data provides clear evidence implicating macrophages in IIL there are some limitations in our model that restrict the direct translation of our findings to human IBD. Firstly, this work was conducted in larval zebrafish in which the intestinal lymphatic vessels are still developing. Although we have ruled out a role for macrophages in developmental lymphangiogenesis, our findings should be validated in an adult model of IBD (Geiger et al., 2013) to ensure that similar mechanisms of inflammatory lymphangiogenesis occur in adult mesenteric lymphatics. However, these studies will be challenging due to the difficulty of live-imaging adult fish and because the anatomy of adult intestinal lymphatics has yet to be described in zebrafish. Second, our immersion model causes widespread inflammation whereas during IBD, inflammation is restricted to the intestine. While it is clear that intestinal inflammation is induced in our model, we cannot rule out the possibility that our IIL phenotype is due to non-intestinal inflammation. There are models of IBD in the fish that induce more localised intestinal inflammation that could be used to further substantiate our findings (Thakur et al., 2014).

The VEGFR pathway is essential for inflammatory lymphangiogenesis in various diseases, including IBD (D'Alessio et al., 2014; Jurisic et al., 2013; Kim et al., 2014). The application of Vegfr inhibitors tivozanib or sunitinib, prevented zebrafish IIL. Treatment with either 150 nm tivozanib or 3 μM sunitinib was sufficient to prevent IIL but at this concentration, developmental lymphangiogenesis in the intestine was inhibited, leading to a loss of the intestinal lymphatics and consequently no ILS could develop following TNBS treatment. Because of this, the concentration of both Vegfr inhibitors was titrated down to a level that allowed developmental lymphangiogenesis to occur but was still sufficient to prevent inflammatory lymphangiogenesis (tivozanib 5 nM, sunitinib 150 nM). These doses were however still 15-fold higher than the inhibitor IC_50_ values for mammalian VEGFR-3; tivozanib 0.24 nM (Nakamura et al., 2006), sunitinib 10 nM (Mendel et al., 2003) suggesting that they are sufficient to inhibit Vegfr signalling and that this pathway has an essential role in our IIL model. However, the question that remained was what tissues or cells provide the VEGFs necessary for IIL to occur?

**Fig. 6.**
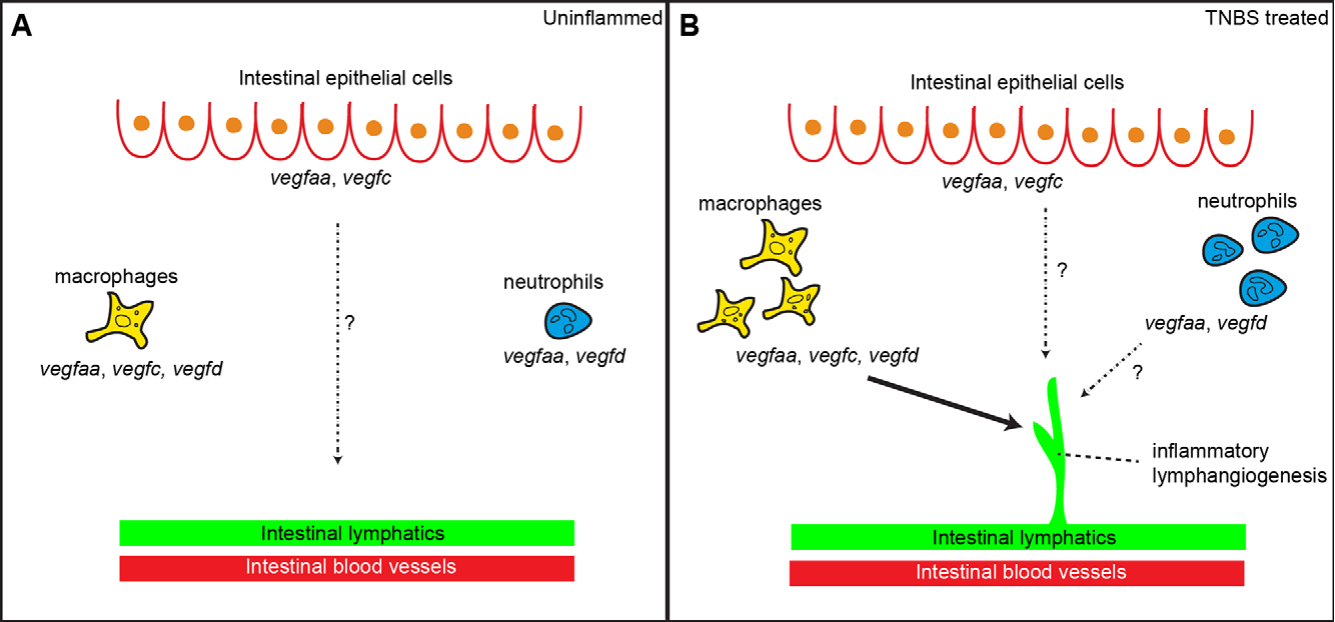
**The potential contributions of macrophages, neutrophils and intestinal epithelial cells towards intestinal inflammatory lymphangiogenesis.** A model showing the potential contributions of macrophages, neutrophils and intestinal epithelial cells towards normal zebrafish intestinal vascular development (A) and zebrafish IIL (B). In the absence of inflammation, *vegfaa*/*c*-expressing zebrafish intestinal epithelial cells may contribute to vascular development in the intestine. In TNBS-treated larvae, *vegfaa/c/d*-expressing macrophages are required to induce inflammatory lymphangiogenesis in the intestine.

Intestinal epithelial cells express *vegfaa* and *vegfc*. This is similar to mammalian intestinal epithelial cells, which have been shown to express VEGF-A and VEGF-C (Griga et al., 2002, 1999b; Joory et al., 2006). Unlike the development of the zebrafish intersegmental vessels that require *vegfaa* expressed in the somites (Lawson et al., 2002; Liang et al., 1998; Nasevicius and Ekker, 2000), the source of Vegfs involved in zebrafish intestinal blood vessel development is still unknown. Given that intestinal epithelial cells express *vegfaa* and *vegfc*, they may contribute to developmental angiogenesis/lymphangiogenesis in the zebrafish intestine by secreting Vegfaa and Vegfc (Fig. 6A). In support of this hypothesis, the formation of the intestinal vasculature is concomitant with the development of the intestine (Isogai et al., 2001; Okuda et al., 2012). However, we found that the expression levels of *vegfc* and *vegfaa* in intestinal epithelial cells remained unchanged following TNBS treatment; while this does not rule out a role for these cells in IIL, it led us to focus on the role of leukocytes in this process.

Mammalian macrophages secrete VEGF-A, VEGF-C, and VEGF-D promoting inflammatory angiogenesis/lymphangiogenesis (Harvey and Gordon, 2012; Szekanecz and Koch, 2007) but their role in IBD-associated lymphangiogenesis is not known. In addition, mammalian neutrophils can secrete VEGF-A and VEGF-D to stimulate tumour-associated angiogenesis (Tazzyman et al., 2013) and skin inflammation-associated inflammatory lymphangiogenesis (Tan et al., 2013), respectively. We show that similar to their mammalian counterparts, zebrafish macrophages express *vegfaa*, *vegfc*, and *vegfd*, while zebrafish neutrophils express *vegfaa* and *vegfd*.

To determine the role of macrophages during IIL we utilised the nitroreductase-mediated cell ablation system (Pisharath and Parsons, 2009). Nitroreductase-mediated cell ablation has been successfully used in zebrafish to remove a variety of cell types including macrophage-lineage cells (Petrie et al., 2014). We found that IIL was suppressed when *mpeg-1*-expressing cells were ablated with metronidazole. In contrast, ablation of neutrophils had no effect on IIL, suggesting that macrophages are the main driver of inflammatory lymphangiogenesis in the intestine. Macrophages are thought to contribute to inflammatory lymphangiogenesis by two mechanisms; the first is via “transdifferentiation” into lymphatic endothelial cells (Kerjaschki et al., 2006; Maruyama et al., 2005) and the second is through the up regulation of pro-lymphatic growth factors such as VEGF-C, either directly via secretion or indirectly by the release of pro-inflammatory cytokines. (Baluk et al., 2005; Cursiefen et al., 2004; Harvey and Gordon, 2012; Kataru et al., 2009; Kim et al., 2009; Kubota et al., 2009; Maruyama et al., 2005; Tan et al., 2014; Zhang et al., 2007). We believe the second mechanism is the predominant one in our model as the lymphatic expansion mediated by macrophages was sensitive to VEGFR inhibitors. Therefore, we propose that the recruitment of pro-lymphatic macrophages to the intestine following colitogenic challenge, contributes to zebrafish IIL (Fig. 6B). In support, reduced macrophage recruitment to the intestine following 5-ASA treatment correlated with decreased zebrafish IIL.

We have previously shown that intestinal inflammation following colitogenic challenge requires the presence of gut microbiota, as treatment with mixtures of the broad-spectrum antibiotics penicillin/streptomycin or ampicillin/kanamycin inhibited the recruitment of leukocytes to the intestine (Oehlers et al., 2011). Metronidazole is an antibiotic, particularly against anaerobic microbes and has been used to treat patients with Crohn's disease (Freeman et al., 1997; Prantera et al., 1996). We found that standard doses of between 5–10 mM of metronidazole that were previously used to conduct nitroreductase-mediated cell ablation in the zebrafish (Pisharath and Parsons, 2009) inhibited IIL in control, non-nitroreductase expressing animals (data not shown), likely due to the antibiotic activity of the pro-drug. However, a reduced metronidazole dose (2.5 mM) ensured that non-nitroreductase expressing animals displayed IIL while still allowing robust ablation of leukocytes using our treatment regimen.

Although macrophage and neutrophil numbers increased throughout the larvae following TNBS treatment (Oehlers et al., 2011), inflammatory lymphangiogenesis was only observed in the intestine. This may be because inflammation was more severe in the intestine than in other areas of the larvae in this model. In support, we previously demonstrated that expression of the inflammatory cytokine, *interleukin 8*, is specifically upregulated in the intestine of larvae exposed to TNBS (Oehlers et al., 2011). In addition, inflammatory angiogenesis was not observed in our model, despite an increase in *vegfaa*-expressing macrophages and neutrophils in the intestine. Intestinal blood vessels start developing at around 2.5 dpf (Isogai et al., 2001) while intestinal lymphatics start developing at around 4 dpf (Okuda et al., 2012). The lymphatic-specific phenotype may therefore be due to the intestinal lymphatic vessels developing later than the intestinal blood vasculature, making lymphatic vessels more susceptible to increased *vegfaa/c/d* expression.

In summary, we have established a novel inflammatory lymphangiogenesis model in zebrafish larvae. We have shown that zebrafish larval macrophages and neutrophils express *vegfaa*, *vegfc*, and *vegfd* and that macrophages contribute to inflammatory lymphangiogenesis in the zebrafish intestine. Our model provides a new platform to investigate the mechanisms of inflammation-driven lymphatic vessel growth.

## MATERIALS AND METHODS

### Zebrafish maintenance

Standard husbandry conditions were utilised to maintain all zebrafish (*Danio rerio*) strains. The transgenic lines used in this study were *Tg(kdrl:EGFP)^s843^* (Jin et al., 2005), *Tg(lyve1b:DsRed2)^nz101^*, *Tg(lyve1b:EGFP)^nz150^* (Okuda et al., 2012), *Tg(mpeg1:EGFP)^gl22^*, *Tg*(*mpeg1:Gal4FF*)*^gl25^* (Ellett et al., 2011), *Tg(lyz:EGFP)^nz117^* (Hall et al., 2007), *Tg(i-fabp:RFP)^as200^* (Her et al., 2004), *Tg(UAS-E1b:nfsB-mCherry)^c264^* (Davison et al., 2007) and *Tg*(-8.*mpx:*KalTA4)*^gl28^*. Zebrafish husbandry and experiments were conducted following the protocols approved by the University of Auckland Animal Ethics Committee.

### Induction of enterocolitis in zebrafish

Intestinal inflammation was induced using 50 μg/ml of TNBS (Sigma-Aldrich, St. Louis, MO, USA) or 0.25% (w/v) of DSS (500,000 MW, Affymetrix, Santa Clara, CA, USA) as previously described (Oehlers et al., 2013). Intestinal inflammation was reduced by co-treatment of 50 μg/ml 5-ASA in E3 (Sigma-Aldrich) with TNBS as previously described (Oehlers et al., 2011). Tivozanib (AVEO Pharmaceuticals Inc, Cambridge, MA, USA) or sunitinib (Sigma-Aldrich) dissolved in DMSO was co-administered with TNBS at 3 dpf.

### FACS

3–7 dpf zebrafish larvae were dissociated as previously described (Covassin et al., 2006; Oehlers et al., 2011). Fluorescent cells were isolated based on forward and side scatter characteristics and fluorescence expression. FACS-assisted cellular isolation was carried out using a FACSAria II (Becton Dickinson, San Diego, CA, USA). FACS analysis experiments were carried out on a BD LSRII (Becton Dickinson, San Diego, CA, USA).

### RNA extraction and RT-PCR

Total RNA from embryos and FACS sorted cells was extracted using Trizol reagent and Trizol LS reagent, respectively using the manufacturer's instructions (Life Technologies, Carlsbad, CA, USA). The cDNA was synthesised using High-Capacity cDNA Reverse Transcription Kit (Life Technologies, Carlsbad, CA, USA) using 1 μg of FACS sorted RNA or 2 μg of total RNA. Primer pairs used for RT-PCR are listed in supplementary material Table S1.

### qPCR

The qPCR was performed using the Platinum SYBR Green qPCR SuperMix-UDG with ROX (Life Technologies, Carlsbad, CA, USA) in an ABI PRISM 7900 HT Fast sequence detection system (Life Technologies, Carlsbad, CA, USA) as previously described (Oehlers et al., 2011). Primer pairs are listed in supplementary material Table S1. All primer pairs spanned an intron to control for any contaminating genomic DNA.

### Live imaging

Confocal images were taken as previously described (Hall et al., 2009) using a Nikon D-Eclipse C1 confocal microscope using z stacks 5 μm apart. Epifluorescence images were taken as previously described (Oehlers et al., 2011) using Leica MZ16A fluorescence stereomicroscope with DFC490 camera. Images were processed using FiJi image processing software (Schindelin et al., 2012), Photoshop CS5 (Adobe, San Jose, CA, USA), and Volocity 5.4 image analysis software (Improvision/Perkin Elmer Life and Analytical Sciences, Shelton, CT, USA).

### Quantification of thoracic duct and intestinal lymphatic sprouts

Thoracic duct formation at 5 dpf was scored as previously described (Astin et al., 2014). ILS development was quantified by taking lateral confocal images of the left intestinal region of *lyve1:DsRed2;kdrl:EGFP* larvae at 7 dpf and measuring the number and total length of ILSs anterior to the boundary of 7th and 8th somites using Volocity 5.4 image analysis software.

### Quantification of neutrophil and macrophage number

Neutrophil and macrophage number was quantified through a fluorescent microscope in the left side of the zebrafish intestine at 7 dpf using *lyz:EGFP* and *mpeg1:EGFP* transgenics, respectively.

### Nitroreductase-mediated cell ablation

For macrophage-lineage and neutrophil ablation, 2.3 dpf embryos were pre-treated with 2.5 mM metronidazole (Sigma-Aldrich). At 3 dpf, embryos were co-treated with either TNBS+2.5 mM metronidazole or 2.5 mM metronidazole only and maintained until 7 dpf. Embryos were kept in the dark throughout.

### Statistical analysis

All statistical analysis and graph plotting was done using GraphPad Prism version 5.0d (GraphPad Software). For single comparisons, unpaired *t*-test (normal distribution) or Mann–Whitney *U*-test were used. For multiple comparisons, one way ANOVA with Dunnett's test (multiple comparisons to control) or Turkey test (compare all data pairs) were used. *P* values of less than 0.05 were considered statistically significant.
